# Complete mitogenome of the entomopathogenic fungus *Metarhizium album* and phylogenetic analysis of Hypocreales

**DOI:** 10.1080/23802359.2021.1914229

**Published:** 2021-05-19

**Authors:** Hui-Hui Sun, Yong-Jie Zhang, Shu Zhang

**Affiliations:** a School of Life Science, Shanxi University, Taiyuan, China; b Institute of Applied Chemistry, Shanxi University, Taiyuan, China

**Keywords:** *Metarhizium album*, mitogenome, Clavicipitaceae, Hypocreales

## Abstract

*Metarhizium album*, with a narrow host range, is an entomopathogenic fungus in the family Clavicipitaceae. Its nuclear genome has been sequenced, whereas its mitogenome is still unknown. In this study, the complete mitogenome of *M. album* was assembled and annotated. This circular mitogenome was 68,425 bp in length and encodes two rRNA genes, 26 tRNA genes, 14 standard protein-coding genes of the oxidative phosphorylation system, and seven intergenic ORFs. A total of 23 introns invaded ten genes, including *atp9* (1 intron), *cob* (2), *cox1* (9), *cox2* (1), *nad1* (1), *nad2* (2), *nad3* (1), *nad4* (1), *nad5* (2), and *rnl* (3). Except for one group II intron (i.e. mL2060), others were all group I introns and involved four subgroups (i.e. IA, IB, IC2 and ID). Phylogenetic analysis based on mitochondrial nucleotide sequences confirmed *M. album* in the family Clavicipitaceae, being closely related to its congeneric *Metarhizium rileyi*.

*Metarhizium album* Petch is an entomopathogenic fungus within the family Clavicipitaceae (Ascomycota: Hypocreales). It was first described as occurring on a leaf-hopper on rice in Sri Lanka (Petch 1931). The fungus has a much more limited host range than its congeneric *Metarhizium anisopliae* and is specific for hemipteran insects, mostly from the family Cicadellidae (Rombach et al. 1987). The nuclear genome of *M. album* was previously reported (Hu et al. 2014). Nevertheless, the mitogenome information of the fungus is still lacking. Herein, we present the complete mitogenome of *M. album*.

*Metarhizium album* strain ARSEF 1941 was recovered from *Nephotettix virescens* (Hemiptera: Cicadellidae) on rice in Roxas, Palawan, Philippines (N10.32, E119.26) and deposited in USDA-ARS Collection of Entomopathogenic Fungal Cultures (https://www.ars.usda.gov/, Melanie Filiatrault, Melanie.Filiatrault@usda.gov). Total DNA was isolated from mycelia and stored at −80 °C at the Laboratory of Microbial Evolutionary Biology at Shanxi University, China. The extracted DNA was fragmented by sonication to a size of ∼280 bp, followed by sequencing on an Illumina Xten platform in 2 × 150 bp reads. Mitogenome was *de novo* assembled from clean reads using NOVOPlasty (Dierckxsens et al. 2017) and then annotated as described previously (Zhang et al. 2017). Introns were named according to a newly suggested nomenclature (Zhang and Zhang 2019).

The mitogenome of *M. album* (GenBank accession: MW448543) was a circular molecule of 68,425 bp with AT content of 73.98%. This mitogenome encoded two rRNAs (*rnl* and *rns*), 26 tRNAs, 14 conserved proteins of the oxidative phosphorylation system (*nad1*-*6*, *4L*; *cob*; *cox1*-*3*, and *atp6*, *8*, *9*), and seven intergenic ORFs. These tRNA genes coded for all 20 standard amino acids. All intergenic ORFs encoded hypothetical proteins.

Ten genes were invaded by a total of 23 introns, including *atp9* (1 intron), *cob* (2), *cox1* (9), *cox2* (1), *nad1* (1), *nad2* (2), *nad3* (1), *nad4* (1), *nad5* (2), and *rnl* (3). Except for one group II intron (i.e. mL2060), all other introns belonged to the group I intron family but fell into four specific subgroups, namely IA (2 introns), IB (11), IC2 (5), and ID (4). Except for one intron (i.e. nad1P636), all other introns contained putative ORFs encoding for ribosomal protein S3, LAGLIDADG or GIY-YIG homing endonucleases, or hypothetical proteins. Evidence for the degeneration of the ORF-lacking intron (nad1P636) was obvious because of frame shifts and stop codon mutations.

Phylogenetic analysis based on mitochondrial nucleotide sequences by the Maximum Likelihood approach confirmed *M. album* as a member in Clavicipitaceae (Figure 1). The fungus was closely related to its congeneric *Metarhizium rileyi* and their clustering received 99% support value. Actually, the phylogenetic tree deduced herein was largely congruent with the tree inferred from nuclear multi-locus analyses (Sung et al. 2008), with all species of the same family clustering as a separate clade (Figure 1).

**Figure 1.**
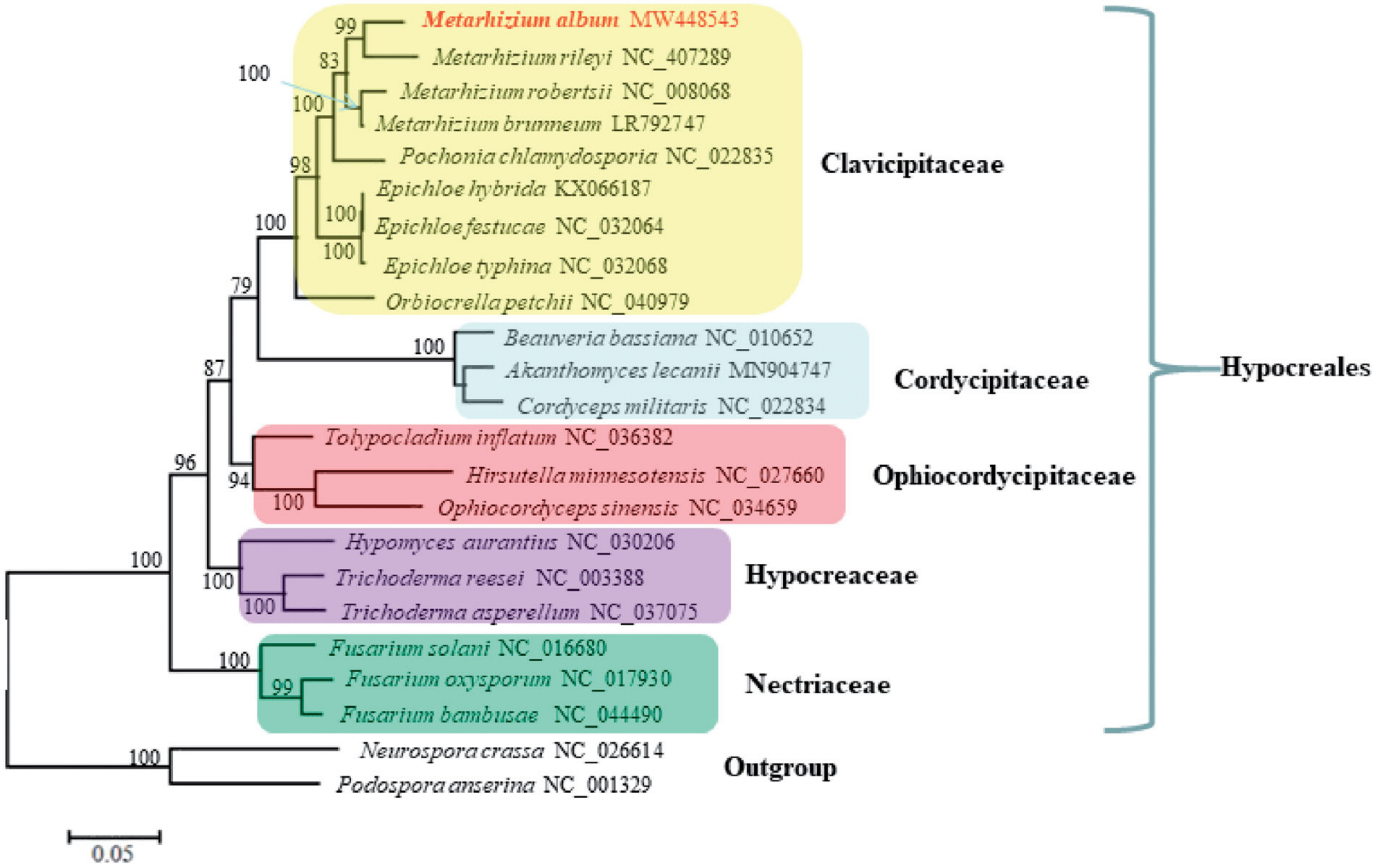
Phylogenetic analysis of Hypocreales species based on mitochondrial nucleotide sequences. We used all species of Clavicipitaceae and representative species of all other families with available mitogenomes in Hypocreales in January, 2021. Two Sordariales species (*Podospora anserine* and *Neurospora crassa*) were used as outgroups. The whole mitogenome sequences (or exonic sequences in cases with alignment difficulties) of these species were aligned and trimmed using the HomBlocks pipeline (Bi et al. 2018), resulting in an alignment of 6911 characters. Phylogenetic reconstruction was performed using the maximum likelihood approach as implemented in RAxML v8.2.12 (Stamatakis 2014). Support values were given for nodes that received bootstrap values ≥ 70%. GenBank accession numbers followed after fungal taxon names.

## Data Availability

The mitochondrial genome sequence data that support the findings of this study are openly available in GenBank of NCBI under the accession no. MW448543 (https://www.ncbi.nlm.nih.gov/nuccore/MW448543).
